# Provider preference for payment method under a national health insurance scheme: A survey of health insurance-credentialed health care providers in Ghana

**DOI:** 10.1371/journal.pone.0221195

**Published:** 2019-08-26

**Authors:** Francis-Xavier Andoh-Adjei, Eric Nsiah-Boateng, Felix Ankomah Asante, Koos van der Velden, Ernst Spaan

**Affiliations:** 1 National Health Insurance Authority, Accra, Ghana; 2 Institute of Statistical, Social and Economic Research (ISSER) University of Ghana, Legon-Accra, Ghana; 3 Radboud Institute for Health Science, Department for Primary and Community Care, Radboud University Medical Centre, Nijmegen, The Netherlands; University of Ghana College of Health Sciences, GHANA

## Abstract

**Background:**

Ghana introduced capitation payment method in 2012 but was faced with resistance from provider groups and civil society organizations for its perceived negative effects on quality care delivery. This study seeks to explore the views of providers to understand their preferred payment method for the various types of services they provide in order to inform the discussion and negotiations during this period of reform. Findings will not only aid the National Health Insurance Authority (NHIA) to improve the implementation arrangements but also provide useful inputs for other low and middle-income countries (LMICs) in their quest to reform their provider payment systems.

**Materials and methods:**

We conducted a cross-sectional survey of 200 credentialed health care providers’ in the three regions of Ghana on providers’ preference for payment method. We administered closed-ended questionnaires employing 5-point Likert scales for measurement of payment method preference. Descriptive and regression analysis were performed to examine healthcare providers’ background characteristics and their association with preferred payment method for primary care.

**Results:**

In general, health care providers prefer the Ghana-Diagnosis-Related Grouping (G-DRG) payment method to fee-for-service and capitation payment methods. Result of bivariate analyses showed that healthcare providers’ preference for payment method for primary outpatient services differed significantly by their region of residence (*p*<0.001). The multinomial logic model showed that being a female (p = 0.013) or healthcare provider in the Volta region (p = 0.008) was significantly associated with health provider preference for G-DRG payment method relative to fee-for-service. Similarly, being a healthcare provider in the Volta region (p = 0.026) or Medical Assistant (p = 0.032) was significantly associated with capitation relative to fee-for-service payment method.

**Conclusion:**

We conclude that the most preferred payment method across all regions is the G-DRG. However, whereas providers in the Volta region are not willing to accept capitation as payment method, this was not the case in Ashanti and Central regions. Capitation payment method as an option for primary care services in Ghana should, therefore, not be ruled out of the discussion.

## Introduction

As demand for health care increases within the context of budgetary constraints, governments are motivated to implement cost-efficiency measures to ensure continuous delivery of quality health care to their populace [1]. At point of service, two efficiency-gains incentives may be applied to contain cost: demand-side incentives to reduce moral hazard and supply-side incentives to induce efficient application of resources [2]. Supply-side, as opposed to demand-side incentives, are deemed a better option because while the demand side incentives, such as co-payments, could impose financial hardship on care seekers, supply-side incentives could induce providers to be cost-conscious in order to control expenditure [2]. One such supply-side incentive is reform of the provider payment system.

Ghana introduced a National Health Insurance Scheme (NHIS) in 2003 after initial pilot in 45 districts between 2002 and 2003. At the start of full scale implementation of the Scheme the NHIA paid its credentialed providers by fee-for-service method. In 2008, the Authority began payment reforms by introducing diagnosis-related grouping (DRG) method for services at all level of care delivery while maintaining fee-for-service for medicines. On realising that the fee-for-service and the DRG payment methods could not help control the observed escalating growth in utilization and claims expenditures, the National Health Insurance Authority (NHIA), which is the regulator, decided to introduce capitation payment for primary care services. Having learned from other experiences that capitation drives down cost [3],[4], serves as critical source of income for providers [5], promotes adherence to guidelines and policies [6] and encourages providers to work better and give health education to patients [7], the NHIA was not oblivious of the fact that capitation is noted to induce reduction in the quantity and quality of care provided [5], encourage skimming on inputs, “dumping” of high risk patients and negatively affect patient-provider relationship [8]. The NHIA was, however, convinced that notwithstanding any un-intended negative effect, with a robust monitoring and evaluation system, implementation of capitation payment could contribute to addressing the cost escalation challenge that was being experienced under the G-DRG and fee-for-service payment methods. Capitation payment was, therefore, introduced as a pilot project in January 2012 in the Ashanti region of Ghana in order to identify un-intended implementation challenges to inform its re-design for step-wise implementation across the country.

The introduction of capitation payment in the Ashanti region was, however, met with resistance from provider groups led by the leadership of their professional associations. The leadership of the faith-based health care provider groups was, however, supportive of the capitation payment method as the Executive Director of Christian Health Association of Ghana (CHAG) was reported to have stated that *“our pledged support (for capitation payment) is premised on the belief that the pilot has the vibrant objective to promote efficiency of health facilities*, *introduce managed competition*, *ensure continuity of care*, *and also ensure cost containment for payers as well as ensuring financial sustainability of the health facilities without compromising quality of care” (*The Ghanaian Times Newspaper of 27/01.2012). Evidence from literature shows that tension between providers and reformers of the payment system tend to slow down the reform process and that such reforms require the intense involvement of all stakeholders, both in the preparation, and implementation of the reform in order to achieve the intended objective [9].

In the midst of a divided opinion of provider groups on the capitation implementation, the desire of the NHIA to reform its payments methods, and the need to accommodate genuine concerns of provider groups to ensure success of the reform agenda, we found it useful to explore the views of providers to understand their preferred payment method for the various types of services they provide. This study would, therefore, provide evidence to inform the discussion and negotiations during this period of reform. Findings will not only aid the NHIA to improve the implementation arrangements but also provide useful inputs for other low and middle-income countries (LMICs) in their quest to reform their provider payment systems.

## Materials and methods

### Study design

We conducted a cross-sectional survey of 200 credentialed health care providers’ in the three regions of Ghana on providers’ preference for payment method. We administered closed-ended questionnaires on the 200 credentialed providers in the three regions (Ashanti, Volta and Central) using face-to-face interview. Data on the main subject of study centered on provider preference for payment method and their attitude towards the capitation payment policy. We also collected data on the socio-demographic characteristics of respondents.

### Research setting

The study took place in three regions of Ghana: Ashanti, Volta and Central regions. Capitation payment policy which is the subject of study was first introduced in the Ashanti region in 2012 and was therefore selected as the “intervention” region for the study. Table 1 below provides the basic socio-demographic and health service/NHIS data on the three regions.

**Table 1:** Basic socio-demographic and health service/NHIS indicators.

Indicator	Ashanti	Volta	Central	National
** a. Socio-demographics Ghana Statistical Service (GSS), 2013**				
Regions’ population relative to national population (%) (2010 PHC)	19.4	8.6	8.9	24,658,823
Economically active population	19.1	8.4	8.6	43.9
Regions’ population employed (%) (2010)	18.6	8.6	8.5	
Self-employed of the employed (%)	65.5	75.3	69.2	64.9
Population density (km^2^)	196	103	224.1	103
Regions’ urban population (%)	60.6	33.7	47.1	50.9
Sex ratio (males/100 females)	94	92.8	91	95.2
Households	1,126,216	495,603	526,764	5,467,136
Average household size	4.1	4.2	4.0	4.4
Regions’ literate population (%)	82.6	73.5	78.2	74.1
** b. NHIS service availability (NHIA, 2013)**				
Number of NHIA District Offices	25	15	13	166
Active NHIS card-bearing members	1,715,174	910,559	866,831	10,145,196
Active members to regional population (%)	34	28	23	35
NHIS-credentialed service providers (2013)	619	321	334	3,832
** c. Health personnel availability (GHS 2013)**				
Percentage share of health professionals	18.2	8.5	8.6	
Percentage share of nurses (Professional)	45.5	53.4	39.3	
Percentage share of nurses (Enrolled)	54.5	46.6	60.7	
Number of Doctors	96	36	26	n/a
Number of Community Health Nurses	157	264	130	n/a
** d. Health service utilization and cost (NHIA 2013)**				
OPD utilization (per member)	2.62	2.63	2.18	2.64
IPD Utilization (per member)	0.17	0.20	0.14	0.18
OPD claims expenditure (GHC per member)	54.52	29.50	42.22	22.14
IPD claims expenditure (GHC per member	35.67	24.33	15.41	41.61

Sources: GSS 2010 Population and Housing Census, 2013. Available at www.statsghana.gov.gh NHIA Annual Report, 2013. Available at www.nhia.gov.gh; NHIA Statistical Bulletin, 2013; GHS Annual Report 2013. Available at www.ghanahealthservice.org

### Population and sampling

The NHIS-credentialed providers constituted the study population from which a sample was drawn for interviews. The sample size was calculated based on number of providers credentialed in 2013, the year when the study was designed; and we used the G-power analysis programme (G* Power 3.1) [10], [11] to determine the appropriate sample size. We assumed an effect size of 0.4, an alpha (α) of 0.05 and beta (1-β) of 0.80; and allocation ratio (N2/N1) of 1.1. The outputs were 200 samples: 95 for group one (Ashanti (intervention) region) and 105 for group two (Central and Volta (control) regions). Based on the number of NHIS-credentialed providers as at the end of year 2013, we then, proportionally, allocated 48% of samples for group two to Volta region and 52% to Central region.

### Data analysis

We performed descriptive analysis of healthcare providers’ socio-demographic characteristics. We then performed chi-square test to determine relationship between providers’ region of residence and their preferred payment method for the various types of services provided under the NHIS in order to understand whether capitation payment would emerge as a preferred payment method for primary out-patients services across all three regions. We also performed a second chi-square test to examine the relationship between health care providers’ characteristics and their preferred payment method. Subsequently, we performed a multinomial logic regression model for healthcare providers’ preference for payment method for outpatients services with three alternatives: fee-for-service (1), G-DRG (2), capitation (3) with fee-for-service being the reference category. Our outcome of interest was “preferred payment method” and the explanatory variables were respondents’ socio-demographic characteristics. Healthcare providers in Ashanti region only were then asked to rate their experiences with the capitation payment policy based on a 5-point Likert scale ranging from “don’t know” (0) to “strongly agree” (4). This was done to estimate mean scores of 21 performance related statements on the capitation policy to assess performance of the policy from the view point of healthcare providers in the Ashanti region where capitation was being piloted. All the analyses were done using Stata version 13 and Excel 2010.

### Ethical considerations

We obtained ethical approval (certified protocol number: UG-ECH 057/13-14) from the University of Ghana Institute of Statistical, Social and Economic Research (ISSER) Ethics Committee for Humanities (ECH). We had earlier sought official permission from the office of the Director-General of the Ghana Health Service (approval letter dated 18/02/2014) to use the NHIS-credentialed health care providers for the study. All respondents who agreed to participate in the survey signed a consent agreement after they were informed that their participation in the study was optional, and their right to privacy was guaranteed.

## Results

### Background characteristics of respondents

One hundred and seventy-three (173) out of 200 healthcare providers sampled for the study participated in the survey (representing 86.5% response rate) of which 87 (50%) were females (Table 2). The average age of respondents was 44 years (SD = 11.33). Seventy-two of the respondents (42%) were in Ashanti region, and 121 (70%) were in the urban setting. Seventy respondents (40%) occupied positions other than medical officer, medical assistant, and Nurse-in-charge. One hundred and thirty-three of the respondents (65%) worked in quasi-government healthcare facilities and 134 (78%) were in the hospital level settings. Respondents’ average age in practice was 11.18 years (SD = 9.62).

**Table 2:** Background characteristics of respondents.

Variable	n (%)
**Age (years)**	
<44	83 (48.0)
44+	90 (52.0)
Mean = 44.30; SD = 11.33	
**Gender**	
Male	86 (49.7)
Female	87 (50.3)
**Region**	
Ashanti	72 (41.6)
Volta	48 (27.7)
Central	53 (30.6)
**Setting**	
Urban	121 (69.9)
Rural	52 (30.1)
**Primary status at facility**	
Medical officer	33 (19.2)
Medical assistant	21 (12.2)
Nurse-in-charge	49 (28.5)
Other (health workers)	70 (40.1)
**Years in practice**	
<11	113 (65.3)
11+	60 (34.7)
Mean = 11.18;SD = 9.62	
**Facility ownership**	
Quasi-government	65 (37.6)
Mission	56 (32.4)
Private	52 (30.1)
**Facility type**	
CHPS	7 (4.0)
Health centre	6 (3.5)
Clinic	21 (12.1)
Maternity home	5 (2.9)
Hospital	134 (77.5)

**Note:** CHPS means Community-Based Health Planning and Services

### Healthcare providers’ preference for mechanism of payment

**Fig 1:**
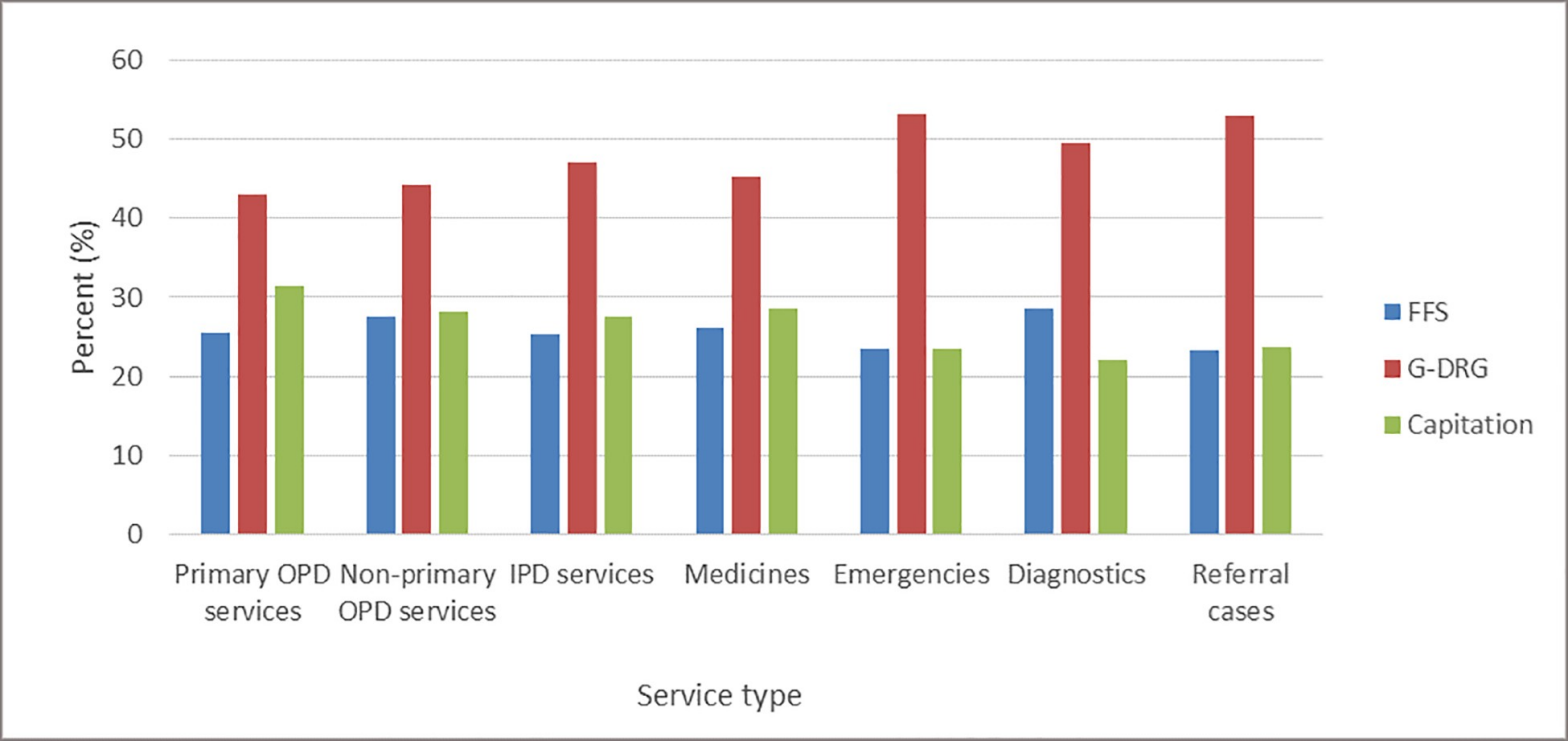
Provider preference for payment method by type of service. Results of the chi-square test showed that healthcare providers’ preference for payment method differed significantly (p<0.001) across all the different types of health care services by region of residence (Table 3). The results further showed that providers in Ashanti region prefer capitation over G-DRG and fee-for-service payments for primary outpatients’ services (47%), non-primary outpatients’ services (39%) and inpatients services (37%). Health care providers in the Volta region preferred G-DRG (79%) to capitation and fee-for-service payments while those in Central showed no clear preference for any particular payment method.

Among the different type of payment methods used by the NHIA, majority of healthcare providers preferred to be paid by the G-DRG method, followed by capitation for all type of services, except diagnostics (Fig 1)

**Table 3:** Healthcare providers’ preferred payment mechanism by region.

Region	Fee-for-service	GDRG	Capitation	Total	Chi-square	p-value
	n (%)	n (%)	n (%)	n (%)		
**Primary outpatient services**						
Ashanti	20 (28.2)	18 (25.4)	33 (46.5)	71 (100.0)	40.48	<0.001
Volta	7 (14.6)	38 (79.2)	3 (6.2)	48 (100.0)		
Central	17 (32.1)	18 (34.0)	18 (34.0)	53 (100.0)		
**Non primary outpatient services**						
Ashanti	21 (30.4)	21 (30.4)	27 (39.1)	69 (100.0)	39.30	<0.001
Volta	6 (12.5)	38 (79.2)	4 (8.3)	48 (100.0)		
Central	20 (37.7)	16 (30.2)	17 (32.1)	53 (100.0)		
**Inpatient services**						
Ashanti	20 (28.2)	25 (35.2)	26 (36.6)	71 (100.0)	30.94	<0.001
Volta	6 (12.5)	38 (79.2)	4 (8.3)	48 (100.0)		
Central	17 (33.3)	17 (33.3)	17 (33.3)	51 (100.0)		
**Medicines**						
Ashanti	22 (31.0)	26 (36.6)	23 (32.4)	71 (100.0)	29.75	<0.001
Volta	6 (12.5)	37 (77.1)	5 (10.4)	48 (100.0)		
Central	17 (32.1)	15 (28.3)	21 (39.6)	53 (100.0)		
**Emergencies**						
Ashanti	21 (30.0)	33 (47.1)	16 (22.9)	70 (100.0)	29.45	<0.001
Volta	6 (12.5)	39 (81.2)	3 (6.2)	48 (100.0)		
Central	13 (24.5)	19 (35.8)	21 (39.6)	53 (100.0)		
**Diagnostics**						
Ashanti	24 (33.8)	29 (40.8)	18 (25.4)	71 (100.0)	38.52	<0.001
Volta	5 (10.4)	41 (85.4)	2 (4.2)	48 (100.0)		
Central	20 (37.7)	15 (28.3)	18 (34.0)	53 (100.0)		
**Referral cases**						
Ashanti	21 (29.6)	32 (45.1)	18 (25.4)	71 (100.0)	26.18	<0.001
Volta	6 (12.5)	39 (81.2)	3 (6.2)	48 (100.0)		
Central	13 (24.5)	20 (37.7)	20 (37.7)	53 (100.0)		

Note: IPD = Inpatient Department; OPD = Outpatient Department; G-DRG = Ghana Diagnosis Related Groupings

### Relationship between healthcare providers’ characteristics and preferred payment mechanism for primary outpatient services

Results of the bivariate analysis showed that healthcare providers’ preference for payment method for primary outpatient services differed significantly by their region of residence (*p*<0.001). However, all other socio-demographic characteristics showed no significant association (Table 4).

**Table 4:** Healthcare providers’ characteristics and preferred payment mechanism for primary outpatient services.

Variable	Fee-for-service	GDRG	Capitation	Total	Chi-square	p-value
	n (%)	n (%)	n (%)	n (%)		
**Age (years)**					3.71	0.295
<44	26 (31.3)	33 (39.8)	24 (28.9)	83 (100.0)		
44+	18 (20.0)	41 (45.6)	31 (34.4)	90 (100.0)		
Mean = 44.30; SD = 11.33						
**Gender**					4.13	0.248
Male	17 (19.8)	41 (47.7)	27 (31.4)	86 (100.0)		
Female	27 (31.0)	33 (37.9)	27 (31.0)	87 (100.0)		
**Region**					40.48	<0.001
Ashanti	20 (28.2)	18 (25.4)	33 (46.5)	71 (100.0)		
Volta	7 (14.6)	38 (79.2)	3 (6.2)	48 (100.0)		
Central	17 (32.1)	18 (34.0)	18 (34.0)	53 (100.0)		
**Setting**					7.68	0.053
Urban	37 (30.6)	49 (40.5)	35 (28.9)	121 (100.0)		
Rural	7 (13.5)	25 (48.1)	19 (36.5)	51 (100.0)		
**Primary status at facility**					18.58	0.099
Medical officer	7 (21.2)	19 (57.6)	6 (18.2)	33 (100.0)		
Medical assistant	2 (9.5))	11 (52.4)	8 (38.1)	21 (100.0)		
Nurse-in-charge	12(24.5)	23 (46.9)	14 (28.6)	49 (100.0)		
Other	22 (31.9)	21 (30.4)	26 (37.7)	69 (100.0)		
**Years in practice**					5.47	0.141
<11	31 (27.4)	52 (46.0)	30 (26.6)	113 (100.0)		
11+	13 (21.8)	22 (36.7)	24 (40.0)	60 (100.0)		
Mean = 11.18;SD = 9.62						
**Facility ownership**					4.31	0.634
Quasi-government	14 (21.54)	29 (44.6)	22 (33.9)	65 (100.0)		
Mission	14 (25.0)	23 (41.1)	19 (33.9)	56 (100.0)		
Private	16 (30.8)	22 (42.3)	13 (25.0)	51 (100.0)		
**Facility type**					3.7	0.988
CHPS	1 (14.3)	3 (42.9)	3 (42.9)	7 (100.0)		
Health centre	1 (16.7)	2 (33.3)	3 (50.0)	6 (100.0)		
Clinic	6 (28.9)	7 (33.3)	8 (38.1)	21 (100.0)		
Maternity home	2 (40.0)	2 (40.0)	1 (20.0)	5 (100.0)		
Hospital	34 (25.4)	60 (44.8)	39 (29.1)	134 (100.0)		

The multinomial logic model revealed that being a female is significantly associated with a 1.32 decrease in the relative log odds of showing preference for G-DRG over fee-for-service payment method (Table 5). However, being a healthcare provider in the Volta region was significantly associated with a 1.89 increase in the relative log odds of showing preference for fee-for-service payment method and a 2.254 decrease in the relative log odds of showing preference for capitation payment versus fee-for-service. Healthcare providers’ setting (urban/rural) showed a positive association at 10% significance level (p = 0.051) for G-DRG relative to fee-for-service. Being a Medical Assistant was also significantly associated with a 2.465 increase in relative log odds of showing preference for capitation relative to fee-for-service.

**Table 5:** Multinomial logic model of providers’ preferred payment mechanism for primary OPD.

Variable	G-DRG			Capitation		
	Coef.	Std. Err.	P>z	Coef.	Std. Err.	P>z
**Age (years)**						
<44	0^b^					
44+	.577	.537	0.282	.856	.555	0.123
**Gender**						
Male	0^b^					
Female	-1.32	.535	0.013	-.873	.561	0.120
**Region**						
Ashanti	0^b^					
Volta	1.89	.717	0.008	-2.254	1.011	0.026
Central	.226	.539	0.674	-.525	.524	0.316
**Setting**						
Urban	0^b^					
Rural	1.113	.569	0.051	.940	.584	0.108
**Primary Status at facility**						
Medical officer	0^b^					
Medical assistant	.997	1.024	0.330	2.465	1.152	0.032
Nurse-in-charge	.832	.770	0.280	.233	.916	0.799
Other	.194	.690	0.779	.168	.788	0.830
**Years in practice**						
<11	0^b^					
11+	-.137	.543	0.801	.169	.540	0.753
**Facility ownership**						
(Quasi)-government	0^b^					
Mission	-.737	.577	0.202	-.193	.574	0.737
Private	.069	.546	0.899	-.292	.587	0.618
**Facility type**						
Health Centre	0^b^					
Clinic	-.726	1.426	0.610	-1.511	1.394	0.278
Maternity home	-.812	1.657	0.624	-1.778	1.809	0.326
Hospital	-.384	1.321	0.771	-.544	1.271	0.668
CHPS	-.009	1.775	0.996	1.124	1.764	0.524
**_cons**	.302	1.515	0.842	.805	1.501	0.592

**Note**: Reference category is fee-for-service; 0^b^: reference variables; G-DRG: Ghana diagnostic related groups; OPD: Out-patient department

### Attitude of health care providers in Ashanti region towards capitation payment policy

Results of analysis on the 21 performance statements on the capitation payment policy in the Ashanti region are summarized in Table 6.

**Table 6:** Mean scores of twenty-one items capitation payment system in Ashanti region (n = 72).

Item no.	Performance statement	Mean	Std. Dev.	Std. Err.	[95% CI]
1	Capitation is a good way of eliminating provider shopping	2.83	0.62	0.07	2.69–2.98
2	Capitation has helped to minimize multiple attendances by NHIS subscribers	2.78	0.58	0.07	2.64–2.92
3	Capitation helps to minimize overcrowding of patients at health facilities	2.36	0.95	0.11	2.14–2.59
4	Capitation can create incentives for providers to reduce quantity of service	2.32	1.00	0.12	2.08–2.56
5	Capitation can help improve the referral system in healthcare delivery	2.74	0.73	0.09	2.56–2.91
6	Capitation will lead to continuity of care	2.72	0.67	0.08	2.56–2.88
7	Capitation will encourage referrals of potentially primary care cases to higher levels of care	2.57	0.81	0.10	2.38–2.76
8	Capitation can create incentives for the prescriber to reduce the quality of service provided to the insured	2.51	0.71	0.08	2.35–2.68
9	Capitation has relieved us of the burden of OPD claims processing and submission	2.07	1.07	0.13	1.82–2.32
10	Capitation has helped to reduce our workload at the OPD.	2.32	0.74	0.09	2.14–2.50
11	Capitation is contributing to efficiency in service delivery	2.19	1.01	0.12	1.96–2.43
12	Capitation provides incentives for us to manage our resources efficiently	2.36	1.02	0.12	2.12–2.60
3	Capitation enables us to do efficient purchasing of items	2.33	0.99	0.12	2.10–2.57
14	Capitation will slow down growth in service utilization	2.72	0.99	0.12	2.49–2.96
15	Capitation can reduce NHIA’s expenditure on primary out-patients claims	2.71	1.27	0.15	2.41–3.01
16	Capitation has helped to eliminate the delayed reimbursement that is experienced under the G- DRG	2.43	0.81	0.10	2.24–2.62
17	Capitation provides a stable income for the provider because of the advance payment.	2.51	0.87	0.10	2.31–2.72
18	Capitation helps us to plan our cash flow better than before	2.39	0.97	0.11	2.16–2.62
19	The capitated rate is enough to cover the primary OPD expenses on the insured clients	1.82	1.09	0.13	1.56–2.08
20	Capitation will reduce the income of the provider	2.51	0.87	0.10	2.31–2.72
21	Capitation will create incentive for the provider to pass on the extra cost of providing care to the insured client	2.36	0.98	0.12	2.13–2.59

Respondents scored most of the statements halfway between “disagree” and “agree” on the 5-point Likert scale. The performance statement *“Capitation is a good way of eliminating provider shopping*” was scored the highest, halfway between “disagree” and “agree” while *“The capitated rate is enough to cover the primary OPD expenses on the insured clients”*, was scored the lowest, halfway between “strongly disagree” and “disagree”. Results of the descriptive statistics also showed that respondents acknowledge that capitation will ensure continuity of care through referral system, slows down growth in utilization and reduce NHIA expenditure.

## Discussion

### Healthcare providers’ preference for payment mechanism and their attitude towards capitation payment method

Different payment methods attract different responses from providers; and provider perception of a payment method could influence their preference for a particular payment method. Findings from our study show that the G-DRG is the most preferred payment method for many providers across the three regions. The data, however, show that the Volta region exhibited high consistency in their preference for the G-DRG and their unwillingness to accept capitation and fee-for-service payment methods. In the Ashanti region, looking at the absolute proportions, capitation payment appears to be the preferred payment method for both primary and non-primary out-patients services as well as in-patients services. However, considering that all three payment methods scored below 50%, it could be said that providers in the Ashanti region neither accept nor reject any of the payment methods in full. The same may be said of the Central region which exhibited similar trend in the analysis. The situation is, however, different in the Volta region where providers reject capitation as a payment method. By implication, one could conjecture that providers in the Volta region are more likely to resist the implementation of capitation payment in the region while those in Ashanti and Central regions are likely to accommodate its implementation. The reasons adduced for the initial resistance to the introduction of capitation payment in the Ashanti region, which attracted widespread, and to some extent, negative media reportage, may have negatively influenced the perception of providers in the Volta region to express their unwillingness to accept capitation as a payment method under the NHIS. The trend in Ashanti suggests that after resisting its introduction at the initial stage, providers in the region may have later recognized and, therefore, come to terms with the positive attributes of capitation payment method. This may be said to have a reflection in the performance statements 1, 2, 5, 6, 14 and 15 (Table 6) that were scored between 2.72 and 2.83 by health care providers. These statements were a mix of positive and negative attributes of capitation payment method and considering that all the negative attributes scored less compared to the positive attributes one may infer that providers’ appreciation of positive attributes outweighs the negative ones.

Earlier studies on the capitation implementation in the Ashanti region [12] and [13] reported of fierce resistance to the capitation policy by medical professionals, civil society organizations and politicians especially during the pre-implementation and early stages of implementations for what they perceived as its potential negative effect on primary care delivery. The initial resistance may be attributed to the reported lack of clarity in the minds of providers between the capitation payment and the G-DRG [14] and as reported in one of Ghana’s national newspapers, the chairman of the Society of Private Medical and Dental Practitioners (SPMDP) admitted that *“lack of education caused the controversy and misunderstanding between the Society of Private Medical and Dental Practitioners in the region and the National Health Insurance Authority”* (Ghana News Agency in The Daily Guide Newspaper of 01/12/2012). This is also confirmed by Dodoo who noted that stakeholder understanding of the capitation payment policy was generally low during pre-implementation and implementation periods which negatively affected their interest and position on the policy [15] leading to the perception that the policy was detrimental to health care providers’ capacity to provide quality healthcare. The same can be deduced from a study by Koduah *et al* [13] who pointed out that perceived and real bureaucratic power which characterized the pre-implementation and implementation stages of the pilot pushed the providers to assert their own powers to “contest and resist various aspects of the policy and its implementation arrangements” without recourse to the technical arguments that are in favour of the capitation payment method. Another key reason for the initial resistance was the perception that the introduction of capitation payment policy in the Ashanti was politically motivated [13], [16],[17] to induce a reduction in the quality of care in the region and thereby cause untimely deaths of potential voters who are perceived to be sympathisers of the then opposition New Patriotic Party.

One may also not rule out the fact that providers’ perceived low capitated rates contributed to the initial resistance as reported by Dodoo [15] and Opoku *et al* [18]. Providers felt that the per capita rate was low [13], non- risk adjusted [19], [13] and lacked clarity on how the per capita rates were calculated [13],[17]. Another plausible reason for the initial resistance was that providers perceived capitation as exposing their facilities to financial risk [12], [18], [19] as may also be inferred from performance statement 19 (Table 6) that was scored about the lowest (1.8) by the health care providers. It is, therefore, plausible to also attribute the seeming preference of providers in the Ashanti region for capitation which scored 46% being the highest to speculation among the public that providers may have identified weaknesses in the design and implementation arrangements and are, therefore, taking advantage to “game” the system. The latter calls for concern and the NHIA may have to critically study the system and improve on the implementation process to avert any such attitude of providers, if indeed the speculation can be substantiated.

### Strengths and limitations

Our study finds strength in the survey design which is relatively suitable for the collection of substantial data on socio-demographics, perception, attitude and behaviour and as such, is widely used in areas such as health and social sciences. We, acknowledge some limitations, one being the lack of in-depth interviews which could have provided opportunity to probe deeper into respondents’ responses to better understand the reasons for their preference of payment method. Another limitation is the comparison among the three regions with some background differences. We, however, also acknowledge the similarities in the demographic and socio-economic characteristics of the two control regions, as well as the similarities in the NHIS/health service data across the three regions which allow for a reasonable level of comparison among the three regions. Findings of the study therefore provide valuable input to enrich the re-design of the capitation payment policy for a smooth implementation.

## Conclusion

This paper sought to explore provider preference for payment method under the National Health Insurance Scheme in Ghana with the view to contributing to the debate on whether or not to migrate from pilot to full-scale implementation of the capitation payment method that attracted resistance of providers through the leadership of their professional associations. Findings from our analysis suggest that, in general, health care providers prefer the G-DRG that was introduced in 2008 to capitation and fee-for-service payment methods. However, the individual level analysis suggests that whereas providers in the Volta region are more likely not to accept capitation as payment method, those in the Ashanti and Central regions neither accepted nor rejected it in totality. We, therefore, conclude that although G-DRG is the most preferred payment method for all service types across all regions, capitation payment as an option for primary care services in Ghana should not be ruled out of the discussion. More education and involvement of health care providers in the (re) design, development and implementation of the policy is, however, recommended.

## Supporting information

S1 Table Raw data on which conclusions were made. (XLSX)

S2 Table Questionnaires administered to respondents. (DOCX)
