# Correction: Provider preference for payment method under a national health insurance scheme: A survey of health insurance-credentialed health care providers in Ghana

**DOI:** 10.1371/journal.pone.0223157

**Published:** 2019-09-23

**Authors:** Francis-Xavier Andoh-Adjei, Eric Nsiah-Boateng, Felix Ankomah Asante, Koos van der Velden, Ernst Spaan

The affiliation for the fifth author is incorrect; the correct affiliation is not indicated. Ernst Spaan is not affiliated with #3, but with: Radboud Institute for Health Science, Department for Health Evidence, Radboud University Medical Centre, Nijmegen, The Netherlands

## References

[1] Andoh-Adjei F-X, Nsiah-Boateng E, Asante FA, Velden Kvd, Spaan E (2019) Provider preference for payment method under a national health insurance scheme: A survey of health insurance-credentialed health care providers in Ghana. PLoS ONE 14(8): e0221195 10.1371/journal.pone.0221195 31449530 PMC6709917

